# Clinical and psychosocial outcomes following correction of supination deformity in obstetrical brachial plexus palsy patients: A retrospective study

**DOI:** 10.1186/s12891-022-05765-0

**Published:** 2022-08-24

**Authors:** Nezar B. Hamdi, Motaz Doubi, Talal B. Abalkhail, Hatan Mortada

**Affiliations:** 1 Department of Orthopedic Surgery, King Faisal Specialist Hospital and Research Center, Riyadh , Saudi Arabia; 2 Division of Plastic Surgery, Department of Surgery, King Saud University Medical City, King Saud University, Riyadh, Saudi Arabia; 3 Department of Plastic Surgery and Burn Unit, King Saud Medical City, Riyadh, Saudi Arabia

**Keywords:** Obstetrical brachial plexus palsy, Supination deformity, Osteotomy, Outcomes, Psychological and psychosocial issues

## Abstract

**Background:**

Forearm supination contracture is the mostAQ common deformity of the forearm following obstetric brachial plexus palsy (OBPP). Supination deformities in OBPP may be corrected by performing forearm osteotomy; however, the high recurrence rate limits patient satisfaction. Apart from the cosmetic impairment of this deformity, there are no previous reports on the clinical and psychosocial outcomes of forearm osteotomy in patients with supination deformities secondary to OBPP. Therefore, our study aimed to assess the clinical, functional, and psychosocial outcomes following forearm pronation osteotomy in OBPP patients with supination deformity.

**Methods:**

This retrospective study was conducted after a chart review of all OBPP sequelae with supination forearm deformity in patients who underwent forearm pronating osteotomy from 2006 to 2018. Data relating to OBPP were gathered, and functional and psychosocial outcomes were assessed using the Disabilities of the Arm, Shoulder, and Hand (DASH) questionnaire through interviews

**Results:**

This study included 60 patients with a mean age of 8.7 years at the time of surgery. A total of 46 patients had lesions involving C5-T1 (76.7%). The mean preoperative supination deformity position was 68.5°, the mean amount of correction was 98.9°, and the mean forearm position was 30.5°, postoperatively. In the DASH assessment scale used postoperatively, 24 patients (42.9%) reported no restrictions on their daily activities, 25 patients (44.6%) believed that their social activities were unaffected, and 20 patients (35.7%) strongly disagreed with feeling less capable or less confident due to arm, shoulder, or hand problems. The factors significantly affecting position at the final follow-up were the amount of correction (p = 0.011), postoperative position (p = 0.005), and degree of pronation achieved (p = 0.02). The amount of correction significantly affected both self-confidence (p = 0.049) and activities of daily living (p = 0.033).

**Conclusion:**

In conclusion, our study showed that the position at the final follow-up, the degree of pronation achieved intraoperatively, and the postoperative position significantly affected the position at follow-up and the outcome assessment. The amount of intraoperative correction was significantly associated with higher self-confidence and normal activities of daily living.

## Introduction

Obstetrical brachial plexus palsy (OBPP) is a common disorder caused by excessive traction of the infant’s head during delivery, stretching of the brachial plexus nerves, and potentially disabling conditions [1]. Such patients were previously followed up until they developed different upper limb deformities and subsequently addressed through soft tissue or bony procedures. Recently, there has been extensive evolution in primary microsurgical repair, which can help improve limb function and decrease the subsequent sequela on muscles and bone [2]. Despite these advancements in the microsurgical repair of OBPP and initial intervention, these patients tend to develop a deformity in most cases [1]. The most common deformity reported was internal rotation contracture of the shoulder. Other deformities include elbow flexion contractures, forearm supination contractures, ulnar deviation of the wrist, and finger deformities. Other less common deformities include winged scapula and forearm pronation contractures [1, 3].

Forearm supination contracture, which is the most common deformity affecting the forearm following OBPP, is a result of a complete OBPP with recovery and spontaneous or post-primary neurosurgical repair of the upper lesion, resulting in a relatively strong biceps brachii muscle and supinator muscles in the presence of a weak or paralyzed pronator muscle. The deformity is flexible and passively pronated at an early stage (less than two years). However, with time, it tends to progress to a fixed deformity due to the contraction of the interosseous membrane, the short biceps brachii, and supinator muscles [4]. The deformity may progress, causing subluxation and dislocation of the distal radioulnar joint, radial head, or radial curvature [4–7].

In addition to cosmetic appearance, developing such a deformity in the forearm significantly impacts the limb’s functional status. Patients and their families also face social and psychological problems due to this condition, which is significant as how the disease affects their daily lives [8].

Multiple surgical approaches and techniques to tackle this deformity have been reported previously, including sectioning of the supinator muscles, the release of the interosseous membrane with biceps brachii muscle rerouting, and bony procedures [7]. Blount et al. was the first to perform a bony procedure, wherein the patient was placed on a cast after closed osteoclasis of the forearm [9]. Zaoussis preferred osteotomy of the radial neck with internal fixation [10]. Gilbert implemented a strategy to approach these patients depending on the clinical picture, whether it was a fixed or flexible deformity, and whether the radial head was dislocated [4, 5, 7, 11].

No previous studies have assessed or quantified the psychosocial outcomes of upper limb function after forearm osteotomy in patients with supination deformity secondary to OBPP. Therefore, our study aimed to assess the clinical, functional, and psychosocial outcomes following forearm pronation osteotomy in OBPP patients with supination deformity.

## Methods and materials

### Study design and setting

From 2006 to 2018, a retrospective chart review of all OBPP sequelae with supination forearm deformities treated with forearm pronating osteotomy by a single surgeon (N.H.) was conducted. The inclusion criteria were patients aged 5 to 30 years with supination contracture due to OBPP and whether or not they were primarily repaired by exploration and grafting. Patients with fair shoulder, elbow, and hand function, regardless of whether they did or did not undergo secondary procedures, including tendon transfers and muscle releases, with a minimum follow-up of 12 months and whose age at last follow-up was more than five years old, were included. Our exclusion criteria were as follows: traumatic brachial plexus injury; poor or no shoulder, elbow, or hand function; follow-up duration of less than one year, and age at last follow-up of fewer than five years.

### Data collection sheet and Ethical approval

Patient charts were reviewed for their preoperative clinical history and postoperative outcomes. The data obtained included the site of OBPP injury (upper, extended, or total), primary surgery, secondary surgery, laterality, pronating osteotomy surgery, age at surgery, age at follow-up, preoperative position, amount of correction, postoperative position, forearm position at the final follow-up, complications including non-union, delayed union, recurrence (defined as a deformity relapsing to the same degree of angle prior to surgery), infection, compartment, nerve injury, regional pain syndrome, hardware failure, revision, and plate removal. The forearm position was measured preoperatively and postoperatively at each clinic visit. The measurement technique involved seating the patient with the shoulder adducted by their side, elbow flexed at 90°, and forearm neutral. Next, the patient was instructed to measure and record their degree of pronation and supination.

After obtaining informed consent, functional and psychosocial outcomes were collected through interviews, including three aspects from the Disabilities of the Arm, Shoulder, and Hand (DASH) questionnaire, which included the following questions: (1) Social: during the past week, to what extent has your arm, shoulder, or hand problem interfered with your normal social activities with family, friends, neighbors, or groups? (2) Activities of daily living (ADL): During the past week, were you limited to your work or other regular daily activities due to your arm, shoulder, or hand problems? (3) Self-confidence: I feel less capable, less confident, or less useful because of my arm, shoulder, or hand problems. In the Arabic-translated DASH version, patients were instructed to circle the number most representative of their condition from 1 to 5 [12]. This study was approved by the Institutional Review Board and Research Ethics Committee of King Faisal Specialist Hospital and Research Center (KFSHRC) in Riyadh, Saudi Arabia. The patients’ medical records were obtained, and data were gathered (reference number 52/26). The contributions of the patients were voluntary, and signed informed consent to use the images for publication was obtained from the patients.

### Statistical analysis

Statistical analyses were performed using SPSS software version 21 (IBM Corp., Armonk, N.Y., USA). Means and standard deviations were obtained for continuous variables, and frequencies and percentages were computed for categorical variables. Continuous variables were assessed using t-tests, while categorical variables were assessed using the chi-square test. A comparison between quantitative variables was performed using Pearson correlation and between two groups of continuous variables using an independent samples t-test. A paired t-test was used to compare the preoperative and postoperative outcomes. One-way ANOVA was used to analyze the correction amount in more than two subgroups (lesions). The outcomes were presented as β coefficients and their respective 95% confidence intervals (95% CIs). The statistical *p*-value of < 0.05 was used to indicate statistical significance.

## Results

This study included 60 patients, of which most were female (*n* = 41, 68.3%). The patients’ mean age was 8.7 years at the time of surgery (range, 2–28 years). The lesion involved the upper trunk (C5, C6) in six cases (10%), an extended upper lesion (C5-C7) in eight (13.3%) cases, and a total lesion (C5-T1) in 46 (76.7%) cases. The right side was affected in 44 patients (73.3%) and the left side was affected in the remaining 16 patients (26.7%). Eighteen patients (30%) underwent primary surgery in the form of nerve transfer and 29 patients (48.3%) underwent secondary surgery ( humeral derotational osteotomy or tendon transfer). The remaining 13 patients (21.7%) did not undergo surgery. The preoperative clinical history and postoperative outcomes of the patients are shown in Table 1. Radial pronating osteotomy with interosseous membrane release was performed in 49 patients (81.7%), and radial and ulnar osteotomies were performed in 11 patients (18.3%). Only nine patients (15%) required revision due to recurrence. Seven patients (11.7%) suffered a hardware failure, and there was one case (1.7%) of non-union. None of our cases was complicated by compartment syndrome, heterotopic ossification, or infection. Preoperatively, the mean forearm position was 68.5 + 25.2° of supination. Postoperatively, the mean forearm position was in 30.5 + 11.7° pronation. The mean intraoperative correction rate was 98.8 + 26.6°. Table 2 summarizes the preoperative and postoperative results of our study samples for the pronating radius osteotomy.

**Table 1:** Demographics and characteristics of the included patients

Variables	Frequency	Percentage (%)
**Gender**		
Male	19	31.7%
Female	41	68.3%
**Site of lesion**^a^		
Upper	6	10.9%
Extended	8	14.5%
Total	42	76.4%
**Laterality**		
Right	44	73.3%
Left	16	26.7%
**Primary surgery (Nerve transfer)**		
Yes	18	30%
No	42	70%
**Secondary surgery (Humeral derotational osteotomy or tendon transfer)**		
Yes	29	48.3%
No	31	51.7%
**Pronating osteotomy surgery**		
Radial osteotomy	49	81.7%
Radial and ulnar osteotomy	11	18.3%
**Reoperation**		
Yes	9	15%
No	51	85%
**Recurrence**		
Yes No	7 53	11.7% 88.3%
**Plate removal**		
Yes No	6 54	10% 90%

^a^ Five patients did not mention the site of the lesion

**Table 2:** An overview of the preoperative and postoperative outcomes following pronating radial osteotomy among our study sample

*Variable*	N	Minimum	Maximum	Mean	Std. Deviation
*Preoperative pronation position*	57	-110	-10	-68.5	25.2
*Degree of correction*	57	30	140	98.8	26.7
*Postoperative pronation position*	57	.00	55	30.5	11.7
*Pronation position at follow up*	51	.00	70	19.7	16.6

**Fig. 1:**
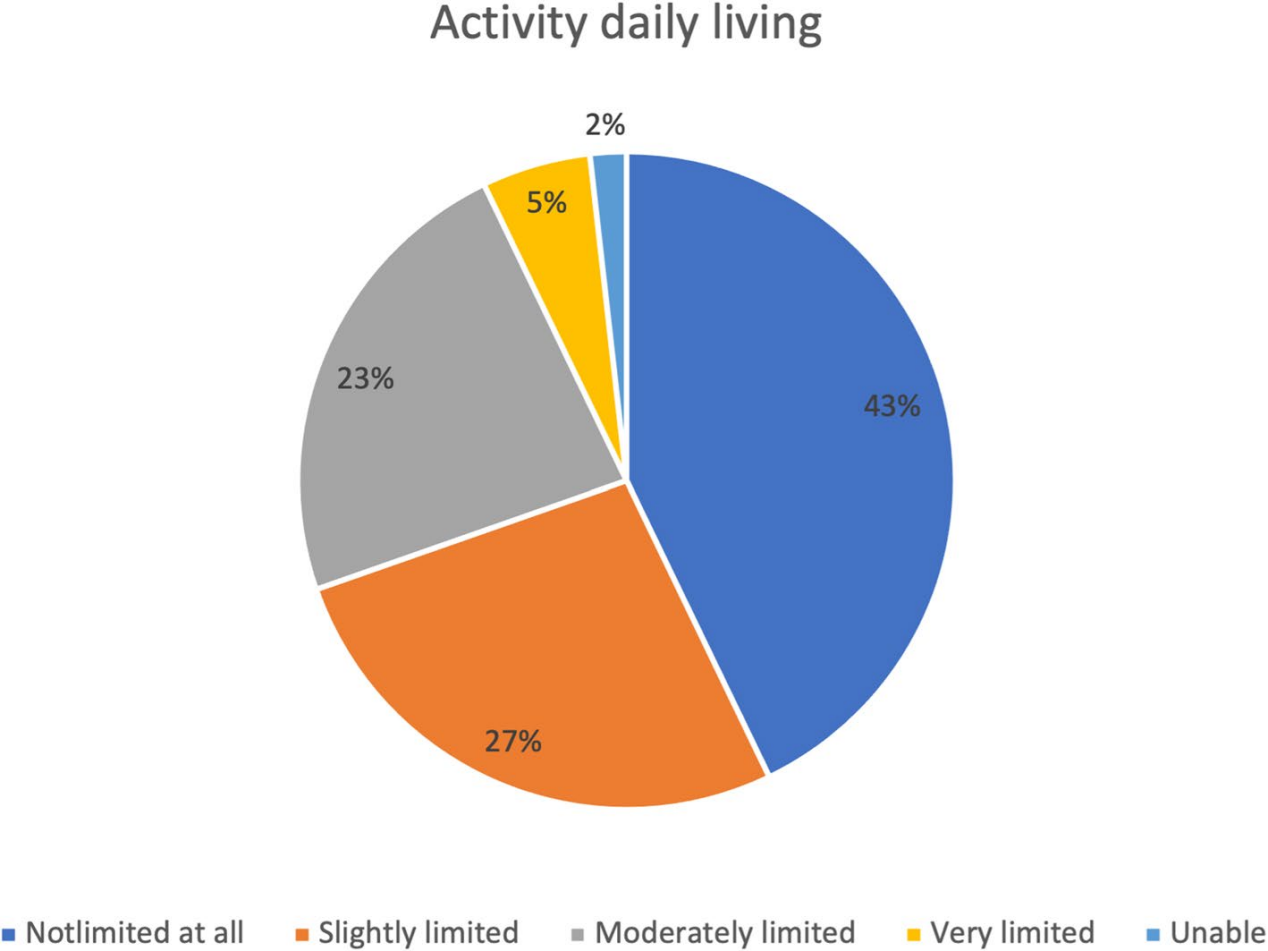
DASH assessment scale for the activity of daily living postoperatively (*n* = 56)

**Fig. 2:**
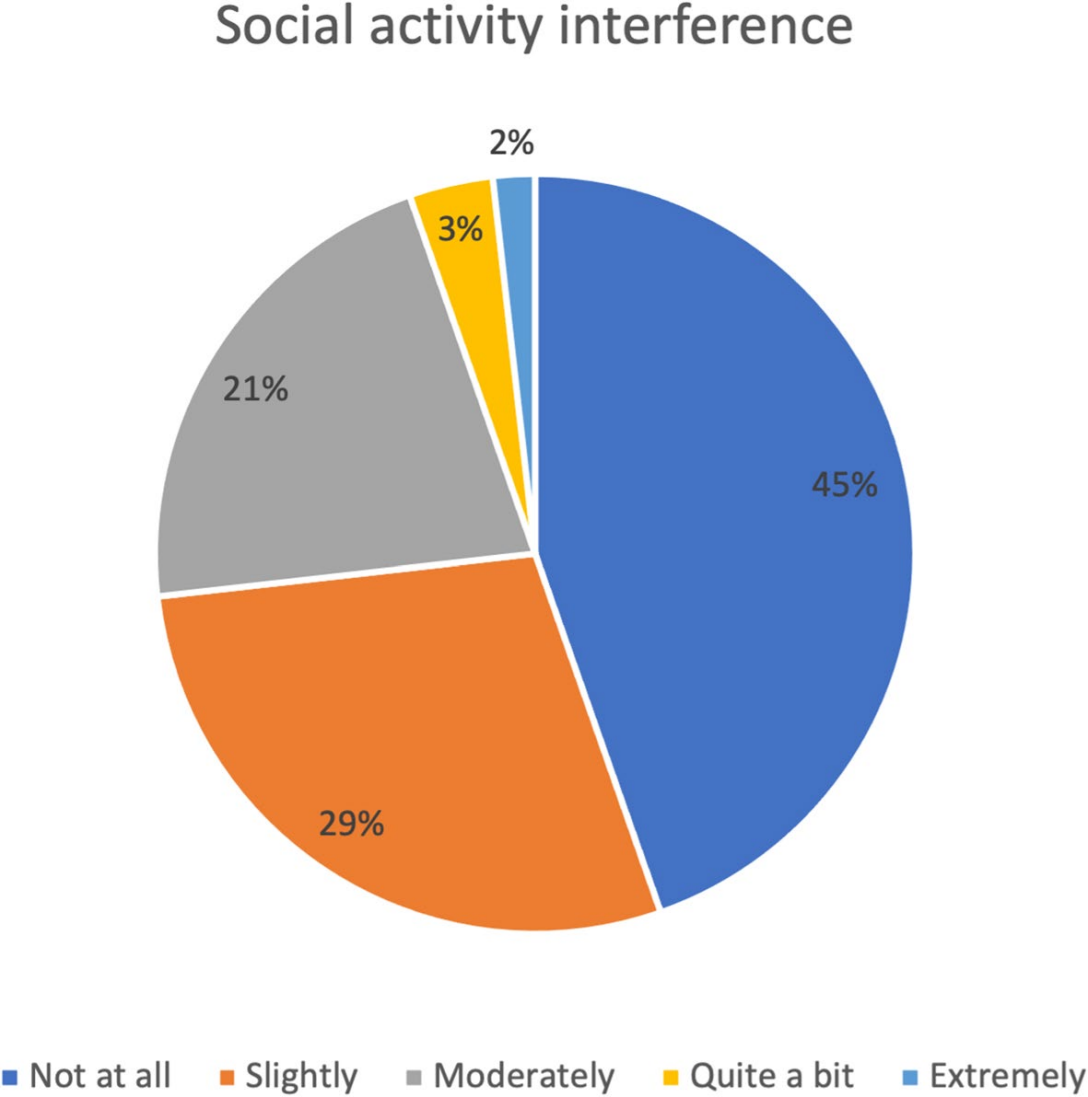
DASH assessment scale for social activity postoperatively (*n* = 56)

**Fig. 3:**
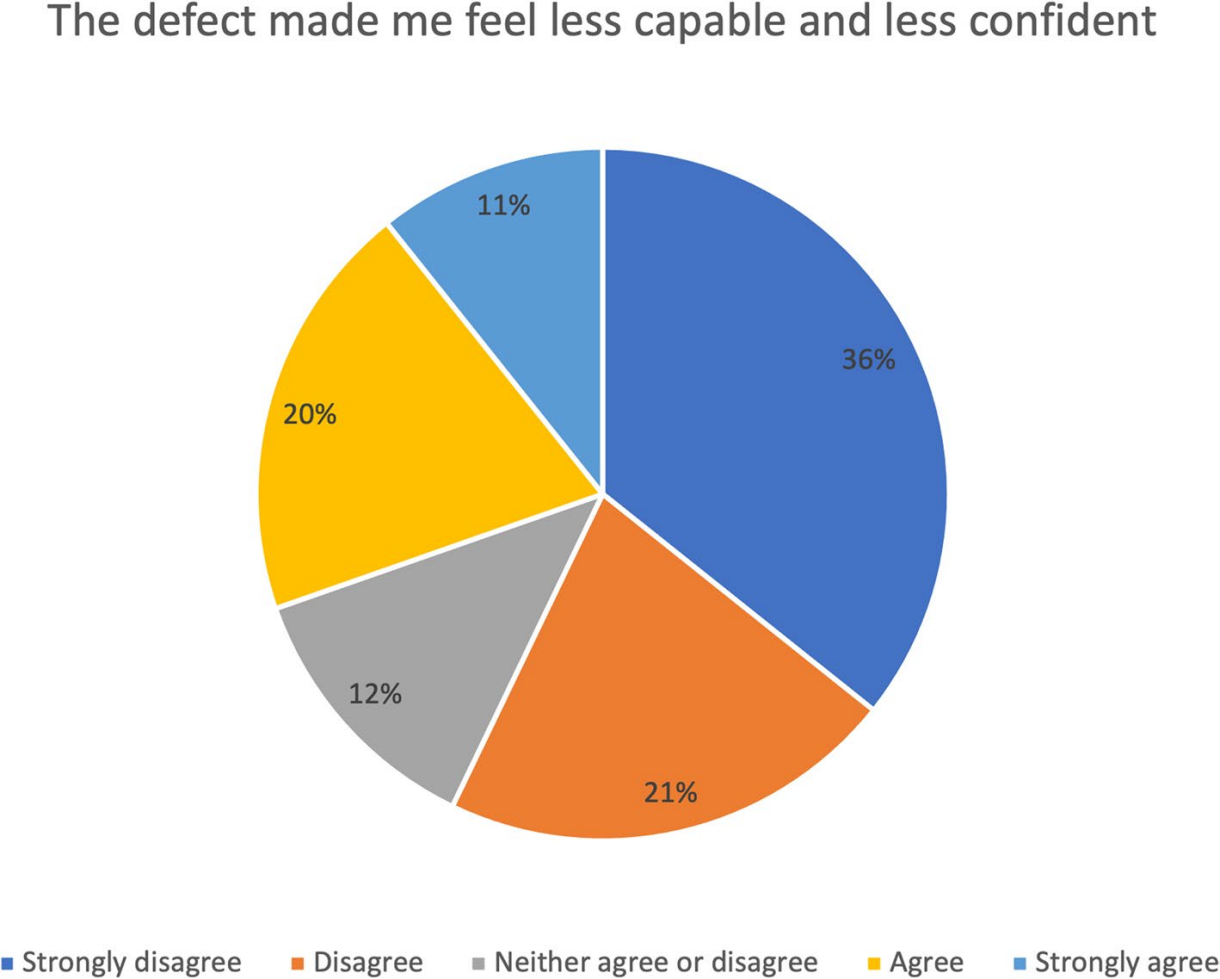
DASH assessment scale, self-confidence: “I feel less capable, less confident, or less useful because of my arm, shoulder, or hand problems” postoperatively (*n *= 56)

Regarding the DASH assessment scale used postoperatively, 24 patients (42.9%) did not experience any limitations in their daily activities, while the functioning of 15 patients (26.8%) was slightly limited (Fig. 1). Regarding social activity, 25 patients (44.6%) believed that it was unaffected, while only 12 patients (28.6%) slightly interfered with their normal social activities (Fig. 2). For the last DASH scale domain, 20 patients (35.7%) strongly disagreed with feeling less capable or less confident because of arm, shoulder, or hand problems. In contrast, six patients (10.7%) strongly agreed (Fig. 3).

An independent Student’s t-test was used to study the effect of multiple variables on the position at follow-up. The position at follow-up was not affected by lesion laterality (*p* = 0.25) or the number of osteotomies (radial osteotomy vs. radial and ulnar osteotomy) (*p* = 0.78). There was no significant difference in the position at follow-up between patients who underwent primary surgery and those who did not (*p *= 0.24), or between patients who underwent secondary surgery (*p* = 0.87), tendon transfer (*p* = 0.44), revision (*p* = 0.76), or plate removal (*p* = 0.49). One-way ANOVA was used to compare the effects of the lesion locations (upper, extended, and total) on the follow-up position, and no statistical significance was found (p = 0.66). Moreover, the age at surgery did not influence recurrence or revision. The mean age at surgery in patients who developed a recurrence was 7.7 years, and for those without recurrence was 8.7 years (*p* = 0.62). The mean age at surgery for patients who underwent revision procedures was 8.2 years, and for those who did not, it was 8.7 years (*p* = 0.79), with a strict recurrence being any supination beyond the neutral position. Position at follow-up significantly influenced self-confidence (*p* = 0.015). However, it did not affect the ADL or social activities. Meanwhile, the amount of correction was found to significantly affect both self-confidence (*p* = 0.049) and ADL (*p* = 0.033) but not social activities.

## Discussion

Forearm supination contractures in patients with OBPP are debilitating. The appearance and function of patients who undergo corrective osteotomy improve significantly. This retrospective analysis examined the outcomes of 60 patients who underwent forearm pronation osteotomy performed by a single surgeon in a tertiary care center, with a mean follow-up period of 3 years. At the last follow-up, the mean position was 19.7 + 16.6° of pronation. Several studies have investigated the etiology of deformities in patients with OBPP. However, the causes of these deformities are still debated. Crouch et al. found that impaired growth [13] was associated with impaired longitudinal muscle growth and shoulder deformity. Weekley et al. tested the hypothesis that OBPP contractures are caused by denervation-induced impairment of elbow flexor muscles. According to the authors, irrespective of muscle imbalance, denervation-induced functional shortening of the elbow flexors leads to variable elbow flexion contractures [14]. As stated by Goh et al., contractures are caused by impaired longitudinal growth of neonatally denervated muscles rather than an imbalance in muscle strength [15].

**Fig. 4:**
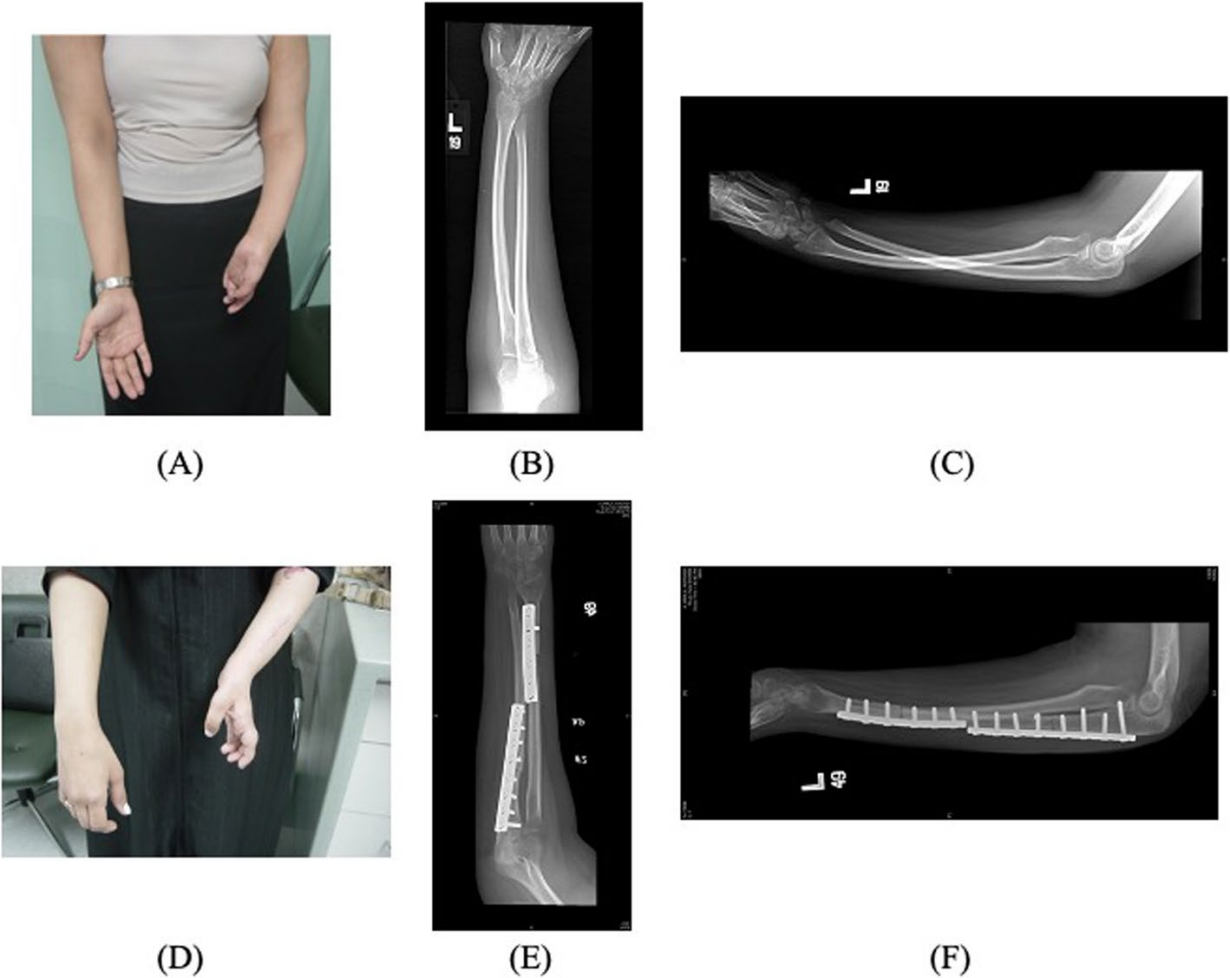
A 25-year-old female with OBPP sequelae with supination deformity of the forearm underwent forearm pronating osteotomy. (a) Left forearm resting position preoperatively, 90° (b) AP radiograph showing incongruent distal radial ulnar joint (DRUJ). (c) Lateral radiograph showing incongruence of DRUJ and bowing of the ulna (**d**) Three months postoperatively, the resting position changed from 90 supinations to 20° of pronation postoperatively (**e**) AP radiograph showing the osteotomy is satisfactorily closed, and the proper position length of the screw (**f**) Lateral view, the length of the screw is adequate and in the proper position

**Fig. 5:**
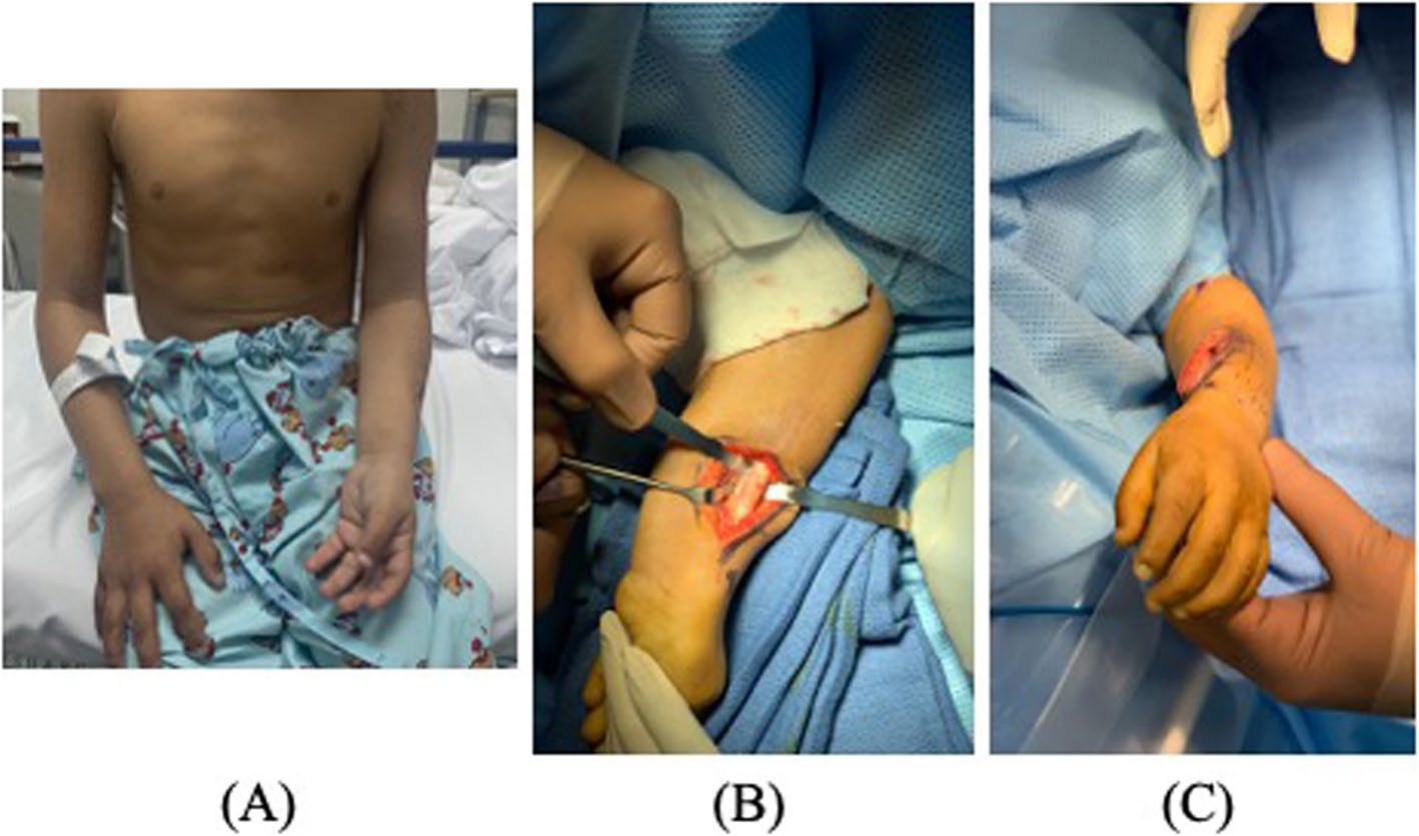
An 8-year-old male with OBPP sequelae and supination deformity of the forearm underwent forearm pronating osteotomy with interosseous membrane release. (a) Left forearm resting position preoperatively, 70° (**b**) Intraoperative image while doing the radial osteotomy (**c**) Immediately after the osteotomy, the resting position changed from 70 supinations to 20° of pronation

Bahm and Gilbert reported findings from patients who underwent surgery for supination contracture following severe obstetric brachial plexus palsy. In 23 patients who underwent open or closed radial osteotomy, the mean postoperative position at four years was 17° in pronation [16]. In another study by Allende and Gilbert, 44 patients who underwent radius osteotomies were followed up for an average of 64.3 months, with a final position at follow-up of 92° [17]. On the other hand, Rolfe et al. studied 14 patients who underwent distal ulnar osteotomy without fixation and subsequent midradial osteotomy with plate fixation. They found a final mean position of pronation of 24° at six months [18]. The intraoperative goal of achieving 30–40° of pronation was determined by a preoperative clinical examination and a dedicated occupational therapist, in addition to the extent of functional impairment caused by the deformity, which prevented the patients from resuming their daily activities. The mean immediate postoperative position of the forearm was 30.5 + 11.7° in pronation, with an average intraoperative amount of correction of 98.8 + 26.6°. Figures 4 and 5 illustrate the findings from some patients included in our study. This is close to what was achieved by Bahm and Gilbert, where the immediate postoperative position was 29° in pronation, and intraoperative derotation was 78° [16]. Allende and Gilbert achieved intraoperative pronation of 114° in 44 patients with radial osteotomies [17]. Kooten et al. studied eight patients whose postoperative resting position was 8° in pronation with a mean intraoperative derotation of 48.75° [19]. Hankins et al. reported an average intraoperative correction of 86° with a final resting position at a final follow-up of 2° in pronation [20]. The different correction rates noted throughout the literature make it challenging to compare the factors influencing the final position at follow-up, which was investigated here and showed that the factors affecting the final position at follow-up were the amount of correction (p = 0.011), postoperative position (p = 0.005), and degree of pronation achieved (p = 0.02). Accordingly, the optimal position for forearm pronation osteotomy varies in the literature.

In this analysis, the age at surgery did not affect the postoperative or final follow-up positions. Additionally, there was no observed age difference between patients who developed recurrence and those who did not (*p* = 0.62), with a recurrence rate of 11.7%. This differs from the findings of a meta-analysis by Metsaars et al., who suggested that recurrence was higher in younger patients with a range of motion of shoulder and hand strength ROM. They also reported a recurrence rate of 41%, which was higher than the rates reported here [21]. Several factors contribute to the recurrence of deformity after forearm osteotomy in patients with supination contractures, including fibrosis and osseous remodeling, along with growth retardation and biceps contractures [21].

In our study, radial pronating osteotomy was performed in 49 patients, while radial and ulnar osteotomies were performed in 11 patients, depending on the power of correction. If the power of correction exceeds 100°, both radial and ulnar osteotomies are required. This aligns with a study by Hutchinson et al. that showed that rotational osteotomy could correct both the radius and ulnar bones to 100°; however, a radial osteotomy can correct only 60° [22]. Most patients underwent radial bone osteotomy with interosseous membrane release, which was our surgical preference. A comparison between the effects of radial osteotomy and radial and ulnar osteotomy on the recurrence rate showed a higher recurrence rate in the radial and ulnar osteotomy group (*n* = 2/11, 18.2%) than in the radial osteotomy group (*n* = 5/49, 10.2%). However, the difference was not statistically significant. This may be because the patients who required radial and ulnar osteotomy in our sample were older and were possibly neglected for social reasons. However, the recurrence rates in patients who underwent radial bone osteotomy with interosseous membrane release were lower in our sample than those reported in the literature. Metsaars et al. did not find a difference between patients treated with mid-distal radius osteotomy, ulnar osteotomy, or combined radius and ulnar osteotomy in active or passive motions, muscle strength, or functional scores at the final follow-up [21]. However, the recurrence rate in that study was 41%. This high recurrence rate could be explained by the fact that they considered any patient with passive pronation of less than 30° to have a recurrence. In contrast, we considered patients who returned to any degree of supination from a neutral degree to recurrence.

Regarding the DASH assessment survey, the position at follow-up was also found to influence self-confidence (*p* = 0.015). However, it did not affect the ADL or social activities. Meanwhile, the amount of correction was found to significantly affect both self-confidence (*p* = 0.049) and ADL (*p* = 0.033) but not social activities. Kooten et al. reported reasonable satisfaction, which was achieved by a neutral postoperative position (8° of pronation) in all their samples based on a simple self-administered survey [19].

### Strength and limitations

Although the primary goal of this study was achieved, some limitations should be mentioned. First, the study sample size was moderate, which can be explained by the low incidence of supination deformities among patients with OBPP. Second, we retrospectively analyzed data from the brachial plexus database rather than prospectively conducting this investigation. Third, it was challenging to obtain the postoperative positions of all patients. Some patients received better rehabilitation than others since many of our included patients lived in faraway regions, and not all of them could follow strict postoperative protocols with close follow-up. Fourth, the DASH assessment was used only postoperatively. Therefore, it was impossible to determine how the procedure affected the overall DASH scores compared with the preoperative status. Therefore, we recommend that future studies utilize the DASH score and compare outcomes preoperatively and postoperatively following forearm pronation osteotomy in patients with supination deformities. Future studies should use the Body Dysmorphic Disorder Assessment Scale. Finally, despite the limited sample size, the comparative aspect of this research and the use of validated upper extremity function assessments provided insights into the impact of ulnar and radial pronation osteotomy for permanent supination contracture due to OBPP. There is an inherent need for additional research on this topic to include a more significant number of patients to improve the validity of our results. In addition, prospective cohort studies are required to confirm the results of this project.

## Conclusion

In conclusion, our study showed that the position at the final follow-up, the degree of pronation achieved intraoperatively, and the postoperative position significantly affected the position at follow-up and the outcome assessment. The amount of intraoperative correction was particularly associated with higher self-confidence and normal ADL. Accordingly, the optimal position for forearm pronation osteotomy varies in the literature.

## Data Availability

The datasets used and/or analysed during the current study are available from the corresponding author on reasonable request.

## Abbreviations

ADL: Activities of daily living
DASH: Disabilities of the Arm, Shoulder, and Hand
KFSHRC: King Faisal Specialist Hospital and Research Center
OBPP: Obstetrical brachial plexus palsy
